# Complete Pathological Response to Neoadjuvant Chemoimmunotherapy in a Patient With Metastatic Intrahepatic Cholangiocarcinoma With High Tumor Mutational Burden

**DOI:** 10.7759/cureus.20187

**Published:** 2021-12-05

**Authors:** Mohammad Abudalou, Eduardo A Vega, Svetlana Kondratiev, Claudius Conrad, Olga Kozyreva

**Affiliations:** 1 Internal Medicine, St. Elizabeth's Medical Center, Boston, USA; 2 Surgical Oncology, St. Elizabeth's Medical Center, Boston, USA; 3 Pathology, St. Elizabeth's Medical Center, Boston, USA; 4 Medical Oncology, Dana-Farber Cancer Institute at St. Elizabeth's Medical Center, Boston, USA

**Keywords:** molecular sequencing, systemic chemotherapy, pathologic complete response, cancer immunotherapy, intrahepatic cholangiocarcinoma

## Abstract

Intrahepatic cholangiocarcinoma (ICC) is an aggressive biliary tract cancer (BTC) with distinct anatomic, molecular, and clinical characteristics. Over the last 10-20 years, ICC has become the focus of increasing concern largely due to its rising incidence and high mortality rates in various parts of the world, including the United States.

Surgery is the only potentially curative treatment option for ICC; however, recurrence rate is high, and prognosis is poor in patients with recurrent disease. The chemotherapy regimen of gemcitabine-cisplatin (GemCis) is still the standard of care for patients with unresectable metastatic ICC. There is limited data regarding pathologic ICC response to palliatively intentioned systemic treatment.

Here, we report a case of a 47-year-old Caucasian male with metastatic ICC microsatellite stable (MSS) and TMB 49 mutation per megabase who achieved complete pathological response with sequential GemCis/nab-paclitaxel and pembrolizumab. This case highlights the effect of sequential neoadjuvant chemoimmunotherapy in a patient with high tumor mutational burden (TMB-H) ICC, emphasizing the importance of molecular testing, which provides valuable information that can be used in clinical practice to better select targeted chemotherapy regimens.

## Introduction

Complete surgical resection remains a potentially curative option for intrahepatic cholangiocarcinoma (ICC); however, only about a third of patients diagnosed with ICC qualify for surgery [1], despite having a poor prognosis with a high rate of recurrence that requires a more aggressive therapeutic intervention [2,3]. Approved treatments for patients with unresectable ICC are limited to chemotherapy regimens tested in heterogeneous study populations of patients with biliary tract cancer (BTC). In a pivotal clinical trial (ABC-02), the standard first-line therapy, consisting of combination therapy with gemcitabine and cisplatin (GemCis), was associated with a median overall survival (OS) of 11.7 months in patients with locally advanced or metastatic BTC, 60% of whom had ICC [4]. More recently, an open-label, single-arm, phase II trial (NCT02392637) demonstrated better survival by the addition of nab-paclitaxel to standard doublet therapy GemCis in patients with BTC (63% of those were ICC), compared to historical controls treated with GemCis alone (ABC-02). Furthermore, 12 patients were able to convert to resectable disease and undergo surgery, two of whom achieved a pathological complete response (pCR). These findings, while promising, still need to be corroborated in the ongoing phase III trial led by the same group (NCT03768414) [5].

Given the poor outcomes linked to ICC, patients and their oncologists are in desperate need of new treatment options. In recent years, the advent of immune checkpoint inhibitors (ICIs) has dramatically changed the landscape of cancer therapy [6]. Among all ICIs applied in clinical studies, anti-programmed cell-death protein 1 (PD-1)/programmed cell-death protein ligand 1 (PD-L1) antibodies are the most successful [7]. Pembrolizumab is an anti-PD-1 antibody approved by the US Food and Drug Administration for the treatment of many cancer types as well as agnostic tumors for patients with microsatellite instability, mismatch repair deficiency, and for which an effective response was recently observed [8,9]. Additionally, the feasibility and safety of neoadjuvant immunotherapy prior to tumor resection have been recently reported. However, limited data are available in TMB-H ICC response to immunotherapy in neoadjuvant setting.

## Case presentation

**Figure 1:**
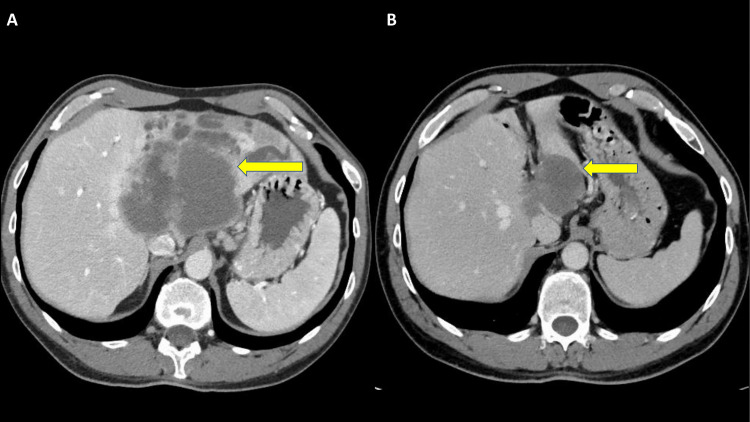
CT of the abdomen. CT scan of a large predominantly hypodense 10x8 cm mass in the central left hepatic lobe at presentation (A). Tumor size decreased to 6.2x4.3 cm after three months of starting immunotherapy (B).

**Figure 2:**
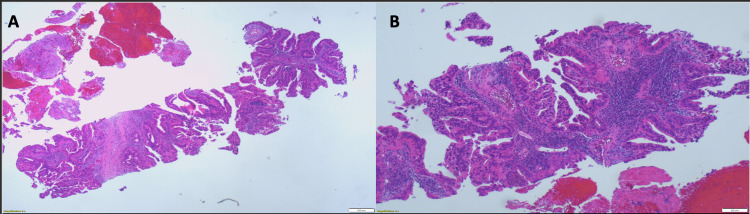
Liver biopsy (H&E). Liver biopsy shows intrahepatic cholangiocarcinoma consisting of infiltrating well-formed glands in an abundant fibrous stroma (A). Malignant glands are lined by cells with varying degrees of atypia and pleomorphism (B). H&E: hematoxylin and eosin stain

**Figure 3:**
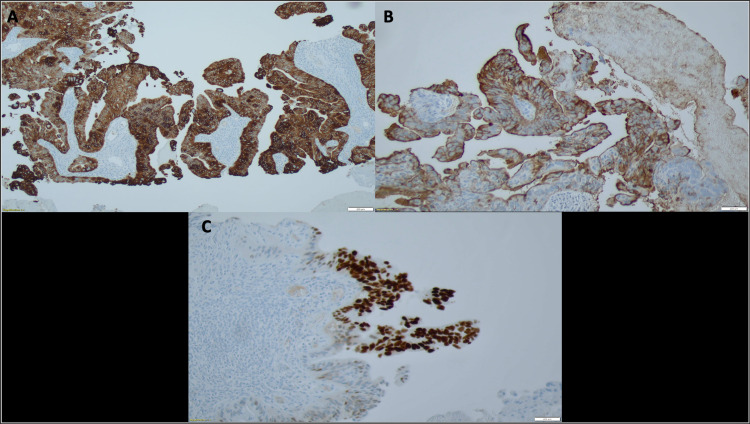
Immunohistochemical stains. Immunohistochemical stains demonstrate diffuse immunoreactivity for CK7 (A), CA19-9 (B), and focal CDX-2 (C).

This case report follows a 47-year-old Caucasian male with a history of recurrent biliary duct stones who was seen by an orthopedic surgeon due to progressive shoulder and back pain. During the evaluation of the patient’s back pain with a spine MRI, a central mass was noted in the liver. A dedicated magnetic resonance cholangiopancreatography (MRCP) and 4-phase liver protocol CT scan revealed a large heterogeneous mass spanning both hepatic lobes, measuring 10 x 8 x 8 cm suspicious for ICC (Figure 1A) and engulfing the left and middle hepatic veins with direct involvement of the right hepatic vein. The left branch of the hepatic artery and the left portal vein were also compromised. The mass caused significant compression, with concern for invasion, of the intrahepatic inferior vena cava (IVC). Also, several enlarged gastrohepatic and right para-aortic lymph nodes were seen. Chest CT showed that the lungs were devoid of any lesions or masses. In the MRCP, the left intrahepatic bile ducts were dilated, but both common hepatic and common bile ducts were intact and non-dilated. At this time, the alpha-fetoprotein (AFP) level was normal, and the carbohydrate antigen (CA) 19-9 tumor marker was elevated at 173 U/mL (negative < 35 U/mL). A biopsy confirmed a moderately differentiated adenocarcinoma (Figure 2). Immunohistochemical stains (IHC) were as follows: CK 7 (Figure 3A) and CA19-9 (Figure 3B) were immunoreactive, CDX2 stain was focally positive (Figure 3C), cytokeratin 20 (CK20) stains showed occasional tumor cells, and thyroid transcription factor 1 (TTF-1) and hepatocyte paraffin 1 (hep Par 1) were negative. As a result, a diagnosis of advanced cholangiocarcinoma was made. On mutational analysis, fibroblast growth factor receptor 2 (FGFR 2) rearrangement and human epidermal growth factor receptor 2 (HER2) mutation were undetectable. No evidence of neurotrophic tropomyosin receptor kinase (NTRK) 1, 2, 3, nor IDH 1 and 2 mutations was found on next-generation sequencing (NGS) analysis. Positron emission tomography (PET) demonstrated an intense fluorodeoxyglucose (FDG) uptake associated with an adjacent 7 mm right peridiaphragmatic lymph node superior to the mass.

He was started on gemcitabine 800mg/m2 and cisplatin 25mg/m2, and paclitaxel 100mg/m2 was added on the second cycle. He completed a total of three cycles of GemCis and nab-paclitaxel without unexpected side effects. NGS (FoundationOne®CDx, Foundation Medicine Inc., Cambridge, Massachusetts) [10] revealed microsatellite stability (MSS) and proficient mismatch repair genes (pMMR), but a TMB-H of 49 mutations. Given that the patient has TMB-H, chemotherapy GemCis plus Nab-p was suspended, and immunotherapy with pembrolizumab 200mg every three weeks was initiated.

**Figure 4:**
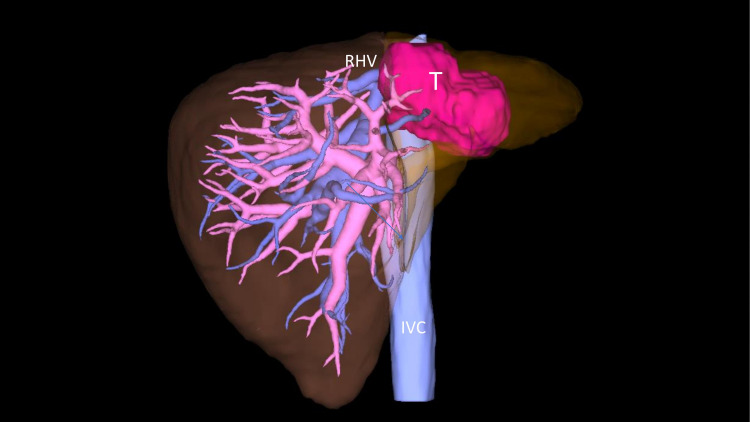
Preoperative planning simulated using pooled data from preoperative CT liver protocol evaluation, on the Synapse Vincent software (Fujifilm, Tokyo, Japan). IVC: inferior vena cava; T: tumor; RHV: right hepatic vein

**Figure 5:**
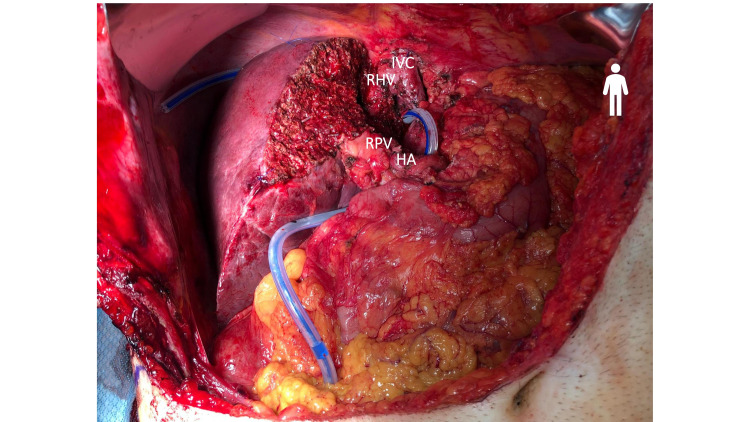
Gross inspection of the liver after left extended hepatectomy with IVC and RHV reconstruction. IVC: inferior vena cava; RPV: right portal vein; HA: hepatic artery; RHV: right hepatic vein

**Figure 6:**
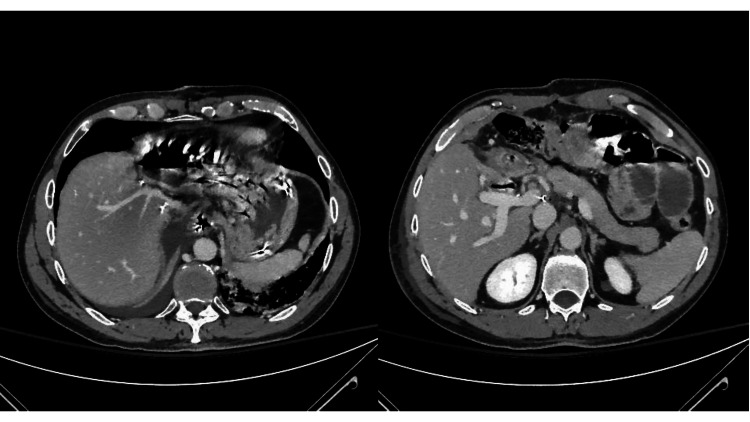
Postoperative CT scan four days after surgery.

**Figure 7:**
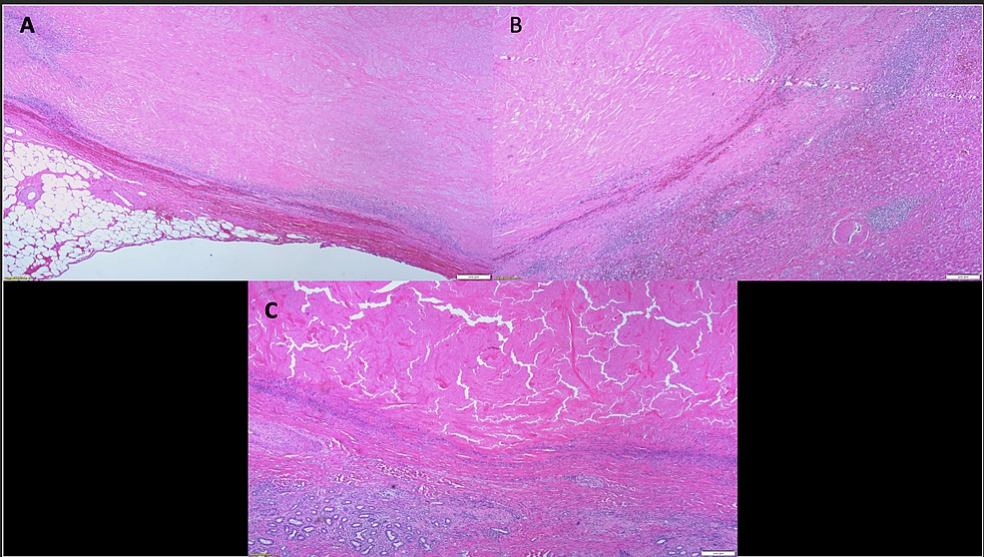
Microscopically, the tumor was completely necrotic (A and B) with adjacent liver parenchyma showing fibrosis, chronic inflammation, and prominent reactive bile duct proliferation (C). No viable tumor was identified.

Follow-up CT scans showed a radiologic response as the tumor size decreased from 10x8.3 cm to 6.2x4.3 cm within three months of starting immunotherapy (Figure 1B). After receiving five cycles of pembrolizumab, the tumor demonstrated a cystic transformation in the following CT, encouraging the hepato-biliary surgery team to reassess him for resection. Standardized future liver volume [11] was estimated at 57.6% (Figure 4). Therefore, the patient underwent open extended left hepatectomy with partial IVC resection, portal and retroperitoneal lymphadenectomy, and right hepatic vein reconstruction (Figure 5, Figure 6). Pathologic analysis confirmed that the patient underwent a complete response to treatment, showing no viable tumor in the specimen (Figure 7). The tumor size was 6.5 cm in its maximum diameter, and resected lymph nodes were devoid of cancer. The patient had an uneventful postoperative course and continues to do well six months after surgery. Two years of adjuvant pembrolizumab is planned.

## Discussion

In this case report, we described a unique patient with metastatic TMB-H ICC who achieved a complete pathological response to sequential chemo- and immunotherapy. From the ABC-02 trial to the recent BILCAP trial, standard chemotherapy has shown limited effectiveness with a modest response rate and OS [4]. Recently triple therapy with GemCis and nab-paclitaxel demonstrated a response rate (RR) up to 45% and OS reaching 19 months [5]. But more important, it was the first trial where two patients with metastatic disease had pCR and eventually underwent surgery. Indeed, pCR for ICC is a rare event with standard neoadjuvant chemotherapy. In the ABC-02 trial, only one patient achieved pCR among 161 patients who received GemCis. Therefore, the limited response rate of cytotoxic chemotherapy has driven the investigation of new alternative treatments for this disease. The relationship between chronic inflammation and the development of BTC has led investigators to harness the immune response through vaccination, adoptive immunotherapy, and checkpoint inhibition as an alternative therapy for BTC [12,13]. Additionally, molecular tumor profiling analysis of targets known to convey susceptibility to specific drugs has been purported to be an effective method for tailoring existing chemotherapeutics agents in order to exploit the specific weakness in individuals’ tumors. NGS and PD-L1 staining has demonstrated that ICC expresses PD-L1, microsatellite sequences, and being TMB-H in 8.1%, 2.5%, and 3.5 %, respectively [14].

Few cholangiocarcinoma cases have reported response to pembrolizumab; however, these tumors were microsatellite unstable or mismatch repair genes (MMR) deficient and only one case achieved pCR with pembrolizumab treatment alone [15-18]. Conversely, our patient was pMMR and MSS but TMB-H. TMB has emerged as an independent predictive biomarker for response to pembrolizumab monotherapy in patients with previously treated, recurrent, or metastatic advanced solid tumors [19,20]. It is FDA-approved for the treatment of TMB-H solid tumors that have progressed following prior chemotherapy and who have no satisfactory alternative treatment options. However, in the front line setting or in combination with conventional cytotoxic therapy, PD-L-1 inhibitors in the treatment of BTC are currently the subject of clinical trials. 

At the time of diagnosis, the tumor was a bulky 10x8.3 cm in size with invasion of vasculature making resection technically impossible. Initially, our patient received triplet palliative chemotherapy employing gemcitabine, cisplatin, and nab-paclitaxel. He achieved partial response after three cycles, however, the tumor was still deemed unresectable. Given TMB-H status, pembrolizumab was pursued as an alternative. After seven cycles, CT still demonstrated a sizable tumor with cystic transformation with the right hepatic vein was still involved; however, it was now amenable for resection with hepatic vein reconstruction. Given the complete response to the treatment with no viable tumor on pathologic examination, no adjuvant chemotherapy was offered. While the patient is planned to continue pembrolizumab for two years based on the duration used in phase 2 Keynote-158 study, it remains a challenge to decide when to stop immunotherapy, which yet needs to be investigated [9]. The combination of pembrolizumab and neoadjuvant chemotherapy resulted in pCR in our case. Our patient received cytotoxic chemotherapy followed by the PD-L1 inhibitor pembrolizumab; therefore, we cannot claim the exact agent or combination that led to pCR.

## Conclusions

The combination of cytotoxic chemotherapy employing GemCis plus nab-paclitaxel followed by pembrolizumab can create a chance for respectability in patients with advanced or metastatic BTC and may even lead to pCR and surgical resectability in this aggressive cancer. This case emphasizes the importance of the information obtained by NGS-based genomic testing in cases of locally advanced or metastatic cancer ICC to guide treatment, given that few standards of care treatment options are available.
